# The role of area‐level socioeconomic disadvantage in racial disparities in cancer incidence in metropolitan Detroit

**DOI:** 10.1002/cam4.6065

**Published:** 2023-05-15

**Authors:** Kristen S. Purrington, Theresa A. Hastert, K. C. Madhav, Mrudula Nair, Natalie Snider, Julie J. Ruterbusch, Ann G. Schwartz, Elena M. Stoffel, Edward S. Peters, Laura S. Rozek

**Affiliations:** ^1^ Department of Oncology Wayne State University School of Medicine Michigan Detroit USA; ^2^ Population Studies and Disparities Research Program Barbara Ann Karmanos Cancer Institute Michigan Detroit USA; ^3^ Department of Internal Medicine Yale School of Medicine, Cancer Outcomes, Public Policy, and Effectiveness Research (COPPER) Center Connecticut New Haven USA; ^4^ Division of Gastroenterology, Department of Internal Medicine University of Michigan Health System Michigan Ann Arbor USA; ^5^ Department of Epidemiology, College of Public Health University of Nebraska Medical Center Omaha Nebraska USA; ^6^ Department of Oncology Georgetown University School of Medicine District of Columbia Washington USA

**Keywords:** geography, racial disparities, social disadvantage, socioeconomic status

## Abstract

**Background:**

Neighborhood deprivation is associated with both race and cancer incidence, but there is a need to better understand the effect of structural inequities on racial cancer disparities. The goal of this analysis was to evaluate the relationship between a comprehensive measure of neighborhood‐level social disadvantage and cancer incidence within the racially diverse population of metropolitan Detroit.

**Methods:**

We estimated breast, colorectal, lung, and prostate cancer incidence rates using Metropolitan Detroit Cancer Surveillance System and US decennial census data. Neighborhood socioeconomic disadvantage was measured by the Area Deprivation Index (ADI) using Census Bureau's American Community Survey data at the Public Use Microdata Areas (PUMA) level. Associations between ADI at time of diagnosis and cancer incidence were estimated using Poisson mixed‐effects models adjusting for age and sex. Attenuation of race‐incidence associations by ADI was quantified using the “mediation” package in R.

**Results:**

ADI was inversely associated with incidence of breast cancer for both non‐Hispanic White (NHW) and non‐Hispanic Black (NHB) women (NHW: per‐quartile RR = 0.92, 95% CI 0.88–0.96; NHB: per‐quartile RR = 0.94, 95% CI 0.91–0.98) and with prostate cancer incidence only for NHW men (per‐quartile RR = 0.94, 95% CI 0.90–0.97). ADI was positively associated with incidence of lung cancer for NHWs and NHBs (NHW: per‐quartile RR = 1.12, 95% CI 1.04–1.21; NHB: per‐quartile RR = 1.37, 95% CI 1.25–1.51) and incidence of colorectal cancer (CRC) only among NHBs (per‐quartile RR = 1.11, 95% CI 1.02–1.21). ADI significantly attenuated the relationship between race and hormone receptor positive, HER2‐negative breast cancer (proportion attenuated = 8.5%, 95% CI 4.1–16.6%) and CRC cancer (proportion attenuated = 7.3%, 95% CI 3.7 to 12.8%), and there was a significant interaction between race and ADI for lung (interaction RR = 1.22, *p* < 0.0001) and prostate cancer (interaction RR = 1.09, *p* = 0.00092).

**Conclusions:**

Area‐level socioeconomic disadvantage is associated with risk of common cancers in a racially diverse population and plays a role in racial differences in cancer incidence.

## 1 INTRODUCTION

Breast, prostate, colorectal, and lung cancers are estimated to account for nearly half of all cancers diagnosed in the United States in 2022 and nearly half of all cancer deaths. While overall incidence of these cancers has declined in the last decade, racial disparities persist. In particular, non‐Hispanic Blacks (NHB) are at increased risk of prostate, colorectal, lung, and certain aggressive breast cancers, and are more likely to be diagnosed at younger ages compared to non‐Hispanic Whites (NHW).^1^ Race is a social construct and, as such, racial disparities in cancer incidence stem from a complex interplay between biological, environmental, social, behavioral, and structural risk factors.^2^, ^3^, ^4^ Work from initiatives such as the Public Health Disparities Geocoding Project has substantially advanced our understanding of how policies such as redlining and mortgage discrimination affect the health of minority populations by creating segregated neighborhoods with adverse socioeconomic status, fewer educational and economic opportunities, increased exposures linked to cancer, and reduced access to high‐quality healthcare.^5^, ^6^, ^7^, ^8^, ^9^, ^10^, ^11^ Despite these advances, there is a clear need for further research to better understand the effect of these structural inequities on racial cancer disparities.^4^, ^11^

The social and built environment have been shown to be as important as individual characteristics for the development of complex health conditions, including cancer.^5^, ^6^, ^7^, ^8^, ^9^, ^10^ NHBs on average have lower socioeconomic status (SES) compared to NHWs, and NHBs with low SES are at higher risk of cancer and poorer cancer outcomes.^12^, ^13^, ^14^ Higher individual‐level SES is associated with increased breast and prostate cancer incidence^15^, ^16^ and lower colorectal and lung cancer incidence.^17^, ^18^ However, individual‐level measures do not capture potentially relevant neighborhood factors such as the physical environment and neighborhood resources.^19^, ^20^ A study of the relative contributions of area‐level and individual‐level SES measures using the VITamins And Lifestyle (VITAL) cohort found that the significant effects of area‐level SES on cancer incidence were only partially explained by individual‐level education and income, indicating that these neighborhood factors confer risk independent of individual‐level SES.^21^ Studies of neighborhood SES and other neighborhood characteristics have not only demonstrated that these factors are associated with cancer incidence, but that individual‐level SES may differentially affect different racial/ethnic groups within different contexts of neighborhood racial composition, residential mobility, and population density.^22^

The goal of the analyses presented in this manuscript was to evaluate the relationship between a comprehensive measure of neighborhood‐level social disadvantage and cancer incidence within the racially diverse population of metropolitan Detroit. We utilized Singh's Area Deprivation Index (ADI), a validated deprivation index that has been associated with poor health outcomes both in the United States and globally,^23^, ^24^, ^25^ but its utility in understanding racial differences in cancer incidence was previously unknown. Specifically, we hypothesized that (1) higher disadvantage would be associated with higher cancer incidence rates and (2) because ADI captures many of the adverse conditions that lead to increased cancer incidence among African Americans, ADI would partially attenuate known relationships between race and cancer incidence. Here we present the results of an analysis linking Singh's ADI with incident cancer data from the Metropolitan Detroit Cancer Surveillance System (MDCSS), a population‐based cancer registry in Wayne, Oakland, and Macomb counties in Michigan to determine whether area‐level deprivation is associated with cancer incidence.

## 2 METHODS

### 2.1 Identification of cancers in metropolitan Detroit (numerator)

All incident primary invasive female breast, prostate, lung, and colorectal cancers among NHW or NHB diagnosed in Wayne, Oakland, and Macomb counties in Michigan between 2012 and2016 (*n* = 42,348) were identified in MDCSS. Excluding individuals diagnosed under 18 years of age, our final sample consisted of 42,332 individuals (12,105 female breast, 6976 colorectal, 11,084 lung, and 12,167 prostate cancers). Molecular subtype was available for breast cancer cases, including 7920 hormone receptor positive (HR+)/HER2‐negative (HER2−) cases, 1370 HR+/HER2‐positive (HER2+) cases, 581 hormone receptor negative (HR−)/HER2+ cases, 1515 HR−/HER2− cases, and 719 cases with unknown subtype (Table 1). This study was conducted under a protocol given concurrence of exemption by the Wayne State University Institutional Review Board on April 23, 2002.

**TABLE 1:** Demographic characteristics of cancer cases and the population of metropolitan Detroit.

	Breast					Colorectal	Lung	Prostate	Population
	Overall	HR+/HER2−	HR−/HER2+	HR+/HER2+	HR−/HER2−				
	*n* (%)	*n* (%)	*n* (%)	*n* (%)	*n* (%)	*n* (%)	*n* (%)	*n* (%)	*n* (%)
Total	12,105	7920	581	1370	1515	6976	11,084	12,167	2,686,466
Age^a^									
18–34	224 (1.9)	95 (1.2)	18 (3.1)	52 (3.8)	47 (3.1)	137 (2.0)	22 (0.2)	1 (0.0)	723,528 (26.9)
35–44	1054 (8.7)	586 (7.4)	64 (11.0)	190 (13.9)	172 (11.4)	301 (4.3)	136 (1.2)	92 (0.8)	426,680 (15.9)
45–54	2607 (21.5)	1644 (20.8)	150 (25.8)	344 (25.1)	359 (23.7)	1168 (16.7)	995 (9.0)	1356 (11.1)	512,106 (19.1)
55–64	3323 (27.5)	2191 (27.7)	171 (29.4)	383 (28.0)	406 (26.8)	1696 (24.3)	2954 (26.7)	4454 (36.6)	490,685 (18.3)
65–74	2706 (22.4)	1907 (24.1)	107 (18.4)	240 (17.5)	314 (20.7)	1661 (23.8)	3607 (32.5)	4478 (36.8)	298,476 (11.1)
75–84	1537 (12.7)	1111 (14.0)	47 (8.1)	120 (8.8)	149 (9.8)	1261 (18.1)	2492 (22.5)	1463 (12.0)	154,822 (5.8)
85+	654 (5.4)	386 (4.9)	24 (4.1)	41 (3.0)	68 (4.5)	752 (10.8)	878 (7.9)	323 (2.7)	80,169 (3.0)
Sex									
Male	0 (0)	0 (0)	0 (0)	0 (0)	0 (0)	3480 (49.9)	5532 (49.9)	12,167 (100)	1,274,113 (47.4)
Female	12,105 (100)	7920 (100)	581 (100)	1370 (100)	1515 (100)	3496 (50.1)	5552 (50.1)	0 (0)	1,412,353 (52.6)
Race									
Non‐Hispanic White	9089 (75.1)	6232 (78.7)	413 (72.1)	1020 (74.5)	904 (59.7)	5009 (71.8)	8378 (75.6)	8408 (69.1)	1,979,087 (73.7)
Non‐Hispanic Black	3016 (24.9)	1688 (21.3)	168 (28.9)	350 (25.5)	611 (40.3)	1967 (28.2)	2706 (24.4)	3759 (30.9)	707,379 (26.3)
ADI quartile									
1	3534 (29.2)	2431 (30.7)	165 (28.4)	378 (27.6)	346 (22.8)	1622 (23.2)	2291 (20.7)	3382 (27.8)	723,361 (26.9)
2	3600 (29.7)	2381 (30.1)	157 (27.0)	421 (30.7)	442 (29.2)	1971 (28.3)	3104 (28.0)	3432 (28.2)	768,044 (28.6)
3	2393 (19.8)	1533 (19.4)	126 (21.7)	286 (20.9)	301 (19.9)	1623 (23.3)	2823 (24.5)	2249 (18.5)	567,826 (21.1)
4	2578 (21.3)	1575 (19.9)	133 (22.9)	285 (20.8)	426 (28.1)	1760 (25.2)	2866 (25.9)	3104 (25.5)	627,235 (23.3)

^a^ Age at diagnosis for cancer cases, age at census for general population.

### 2.2 Metropolitan Detroit population count data by geographic region (denominator)

We obtained population counts for each of the 27 Public Use Microdata Areas (PUMAs) contained entirely within Wayne, Oakland, and Macomb counties in Michigan stratified by age (by year of age for individuals 18+ years old), race (African American, white), and sex (male, female) from United States 2010 decennial census Integrated Public‐Use Microdata Samples (IPUMS) (https://usa.ipums.org/usa/). PUMAs are geographic units used by the United States Census for providing statistical and demographic information, containing at least 100,000 people, and these units represent the smallest geographic units for which population count data are available stratified by race, age, and sex.

### 2.3 Geocoding residential addresses for cancer cases

Residential addresses for MDCSS‐identified cases at the time of cancer diagnosis were geocoded using the Federal Information Processing Standard (FIPS) convention to identify census tract for each cancer case. To achieve comparability in geographic region for cancer cases and population counts, we then identified the census tracts contained within each metropolitan Detroit PUMA unit using the “2010 Census Tract to 2010 PUMA Relationship File” (https://census.gov/program‐surveys/geography/guidance/geo‐areas/pumas.html).

### 2.4 Area‐level deprivation index calculation

We calculated ADI at the census‐tract level for each of 1166 census tracts in Wayne, Oakland, and Macomb counties in Michigan. Census tract geocodes were obtained from GIS Open Data (2010 Census Tracts v17a, https://gis‐michigan.opendata.arcgis.com/datasets/). Michigan's 5‐year estimates (2009–2013) from the Census Bureau's American Community Survey (ACS) were abstracted from the US census. The census‐derived indicators used in the calculation of ADI include educational distribution (percentage of the population with less than 9 years and with 12 or more years of education), median family income, median home value, median gross rent, median monthly mortgage, income disparity, unemployment, percent employed person in white‐collar occupation, percent families below poverty, percent population below 150% poverty threshold, single‐parent household rate, homeownership rate, percent household without a telephone, percent household without a motor vehicle, percent occupied housing units without complete plumbing, and household crowding. The 17 US census indicators were multiplied by the Singh's coefficients (factor weights) and summed to obtain the base score for all census tracts in the catchment area.^26^, ^27^ Each base score was standardized by dividing the difference between the individual census tract base score (*b*) and census tract mean (*p*), by census tract standard deviation (*S*_*p*_).

$$\text{Standard base}\left(j\right)=\frac{b-p}{S_{p}}\;j=1,2,\ldots ,k$$

where *j* represents the *j*th census tract and *k* is the total number of census tracts in the catchment area. Standardized values were adjusted to a base mean of 100 and a standard deviation of 20^26^:

$$\mathrm{AD}I_{j}=\left({\text{Standard base}}_{j}+100\right)*20$$

Higher ADI scores correspond to higher socioeconomic disadvantage. To map census tract‐level ADI scores to PUMAs, we calculated the weighted median of census‐tract ADI scores within each PUMA, with weights corresponding to the population size of each census tract. PUMA‐level weighted median scores were then linked to the MDCSS‐identified cancer cases and population‐level count data.

### 2.5 Statistical analyses

All statistical analyses were conducted in R (https://cran.r‐project.org/). To aid with interpretability, weighted median ADI was categorized into quartiles, where quartile boundaries were calculated using the overall study population ADI distribution across all metropolitan Detroit census tracts. Univariable associations between demographics, race, and ADI were examined using chi‐squared tests and Wilcoxon rank sum tests for categorical and continuous variables, respectively. Age‐ and sex‐adjusted incidence rates were calculated using the “rate” function in the “popEpi” R package, where person‐years (PY) were calculated as ${\text{population size}}_{\mathrm{age}-\mathrm{sex}\;\text{stratum}}\times 5\;\text{years}$ and weights were calculated as the sum of the total PY per age‐sex stratum. Incidence rates were calculated separately for breast cancer, colorectal cancer, prostate cancer, and lung cancer, stratified by race (NHW and NHB). Risk ratios were calculated using a Poisson mixed‐effects model to account for group‐level PUMA effects as implemented by the “glmer” function in the “lme4” R package adjusting for age and sex, as appropriate. ADI quartile was operationalized as a categorical variable for the majority of analyses, using ADI quartile 1 as the reference (Tables 2 and 3). Associations with a significant *p*_trend_ are summarized in the results section for simplicity using ADI as an ordinal variable. We used the “mediation” package to quantify the proportion of the total association of race on cancer incidence attenuated by inclusion of ADI in our multivariable models. Post hoc analysis evaluating ADI as an effect modifier of the relationship between race and cancer incidence was conducted by including a race × ADI interaction term in these models. Maps were generated using GeoDa software.^28^

**TABLE 2:** Age‐ and sex‐adjusted incidence rate and rate ratios by ADI quartile and cancer site for non‐Hispanic Whites and non‐Hispanic Blacks.

	Non‐Hispanic White		Non‐Hispanic Black	
	IR^a^ (95% CI)	RR (95% CI), *p*_trend_	IR^a^ (95% CI)	RR (95% CI), *p*_trend_
Breast cancer: overall				
ADI: Q1	177.7 (171.7, 183.9)	1.00 (ref)	189.7 (151.1, 238.3)	1.00 (ref)
ADI: Q2	171.2 (165.1, 177.5)	0.93 (0.83, 1.03)	186.1 (170.5, 203.2)	1.00 (0.85, 1.19)
ADI: Q3	161 (153.9, 168.4)	0.85 (0.76, 0.96)	181.1 (163.3, 200.8)	0.94 (0.79, 1.12)
ADI: Q4	164.6 (153.2, 176.8)	0.78 (0.67, 0.91), 0.00028	159.9 (152.5, 167.6)	0.87 (0.75, 1.02), 0.0017
Breast: HR+, HER2−				
ADI: Q1	124.2 (119.2, 129.4)	1.00 (ref)	110.9 (77.6, 158.4)	1.00 (ref)
ADI: Q2	116.7 (111.7, 121.9)	0.91 (0.80, 1.02)	106.8 (95.0, 120.1)	1.11 (0.88, 1.40)
ADI: Q3	106.5 (100.8, 112.6)	0.81 (0.71, 0.93)	101.5 (88.3, 116.7)	1.00 (0.79, 1.28)
ADI: Q4	117.0 (107.5, 127.4)	0.79 (0.67, 0.94), 0.00079	90.5 (85.0, 96.4)	0.96 (0.77, 1.19), 0.056
Breast: HR−, HER2+				
ADI: Q1	8.4 (7.1, 9.8)	1.00 (ref)	7.2 (3.7, 13.9)	1.00 (ref)
ADI: Q2	7.4 (6.2, 8.8)	0.85 (0.66, 1.10)	9.4 (6.4, 13.8)	1.01 (0.48, 2.16)
ADI: Q3	8.2 (6.7, 10.0)	0.92 (0.70, 1.22)	11.1 (7.3, 16.7)	1.16 (0.54, 2.50)
ADI: Q4	6.9 (4.9, 9.7)	0.80 (0.54, 1.19), 0.32	8.7 (7.1, 10.6)	0.99 (0.49, 1.98), 0.87
Breast: HR+, HER2+				
ADI: Q1	19.0 (17.1, 21.1)	1.00 (ref)	16.6 (10.3, 26.8)	1.00 (ref)
ADI: Q2	19.4 (17.4, 21.6)	0.97 (0.83, 1.14)	25.7 (20.4, 32.3)	1.40 (0.85, 2.30)
ADI: Q3	20.1 (17.7, 22.8)	0.97 (0.82, 1.16)	17.9 (13.1, 24.6)	0.95 (0.55, 1.63)
ADI: Q4	19.7 (16.0, 24.3)	0.93 (0.73, 1.19), 0.60	17.3 (15.0, 19.9)	0.93 (0.58, 1.49), 0.024
Breast: HR−, HER2−				
ADI: Q1	16.0 (14.2, 17.9)	1.00 (ref)	46.7 (34.7, 62.6)	1.00 (ref)
ADI: Q2	18.7 (16.8, 20.9)	1.12 (0.91, 1.38)	35.8 (29.5, 43.5)	0.71 (0.51, 0.99)
ADI: Q3	17.3 (15.1, 19.8)	1.02 (0.82, 1.28)	37.9 (30.4, 47.3)	0.74 (0.53, 1.05)
ADI: Q4	15.4 (12.2, 19.4)	0.88 (0.64, 1.20), 0.54	31.6 (28.4, 35.1)	0.63 (0.47, 0.85), 0.010
Colorectal cancer				
ADI: Q1	41.3 (39.3, 43.5)	1.00 (ref)	48.8 (37.5, 63.4)	1.00 (ref)
ADI: Q2	47.4 (45.2, 49.8)	1.13 (1.03, 1.25)	59.4 (52.6, 67.1)	1.14 (0.85, 1.54)
ADI: Q3	55.3 (52.4, 58.4)	1.32 (1.19, 1.46)	67.1 (58.8, 76.6)	1.31 (0.97, 1.77)
ADI: Q4	45.9 (41.7, 50.4)	1.04 (0.91, 1.19), 0.084	65.9 (62.3, 69.7)	1.39 (1.05, 1.84), 0.010
Lung cancer				
ADI: Q1	60.5 (58.0, 63.1)	1.00 (ref)	33.3 (24.3, 45.5)	1.00 (ref)
ADI: Q2	77.8 (74.9, 80.8)	1.23 (1.04, 1.46)	65.4 (58.2, 73.4)	1.99 (1.45, 2.74)
ADI: Q3	105.3 (101.2, 109.5)	1.67 (1.40, 2.00)	90.4 (80.3, 101.8)	2.58 (1.88, 3.54)
ADI: Q4	91.0 (85.1, 97.3)	1.28 (1.06, 1.55), 0.0028	101.0 (96.5, 105.7)	3.18 (2.36,4.29), <0.0001
Prostate cancer				
ADI: Q1	172.5 (166.6, 178.7)	1.00 (ref)	277.4 (234.4, 328.4)	1.00 (ref)
ADI: Q2	168.2 (162, 174.6)	0.95 (0.86, 1.04)	289.8 (265.8, 315.9)	1.11 (0.85, 1.45)
ADI: Q3	159 (151.7, 166.7)	0.88 (0.80, 0.98)	278.9 (252.5, 308.1)	1.10 (0.84, 1.45)
ADI: Q4	151.9 (141.1, 163.5)	0.81 (0.72, 0.92), 0.00048	279 (267.8, 290.6)	1.21 (0.95, 1.55), 0.173

^a^ Incidence rate per 100,000 person‐years adjusted for age and sex (lung and colorectal only).

**TABLE 3:** Associations between race and cancer incidence before and after adjustment for ADI.

	Non‐Hispanic White	Non‐Hispanic Black	Age‐ and sex‐adjusted		Age‐, sex‐, and ADI‐adjusted	
	IR^a^ (95% CI)	IR^a^ (95% CI)	Rate ratio (95% CI)	*p*‐value	Rate ratio (95% CI)	*p*‐value
Breast	169.8 (166.3, 173.3)	165.4 (159.5, 171.6)	0.96 (0.92, 1.00)	0.052	1.03 (0.98, 1.08)	0.31
HR+, HER2−	116.5 (113.7, 119.5)	93.2 (88.8, 98.0)	0.79 (0.74, 0.83)	<0.0001	0.83 (0.78, 0.89)	<0.0001
HR−, HER2+	7.8 (7.1, 8.6)	8.9 (7.1, 7.6)	1.13 (0.94, 1.35)	0.20	1.22 (0.97, 1.51)	0.086
HR+, HER2+	19.3 (18.2, 20.6)	18.6 (16.7, 20.8)	0.94 (0.83, 1.06)	0.34	1.01 (0.87, 1.17)	0.88
HR−, HER2−	17.0 (16.0, 18.2)	33.7 (31.0, 36.5)	1.96 (1.76, 2.17)	<0.0001	2.14 (1.88, 2.42)	<0.0001
Colorectal	46.6 (45.3, 48.0)	62.6 (59.8, 65.5)	1.33 (1.26, 1.40)	<0.0001	1.28 (1.20, 1.36)	<0.0001
Lung	78.3 (76.7, 80.1)	88.3 (84.9, 91.8)	1.11 (1.07, 1.16)	<0.0001	0.94 (0.89, 0.99)	0.024
Prostate	165.1 (161.6, 168.7)	273.3 (264.4, 282.4)	1.64 (1.58, 1.71)	<0.0001	1.72 (1.64, 1.81)	<0.0001

^a^ Incidence rates adjusted for age and sex (colorectal and lung only) per 100,000 persons per year.

## 3 RESULTS

**FIGURE 1:**
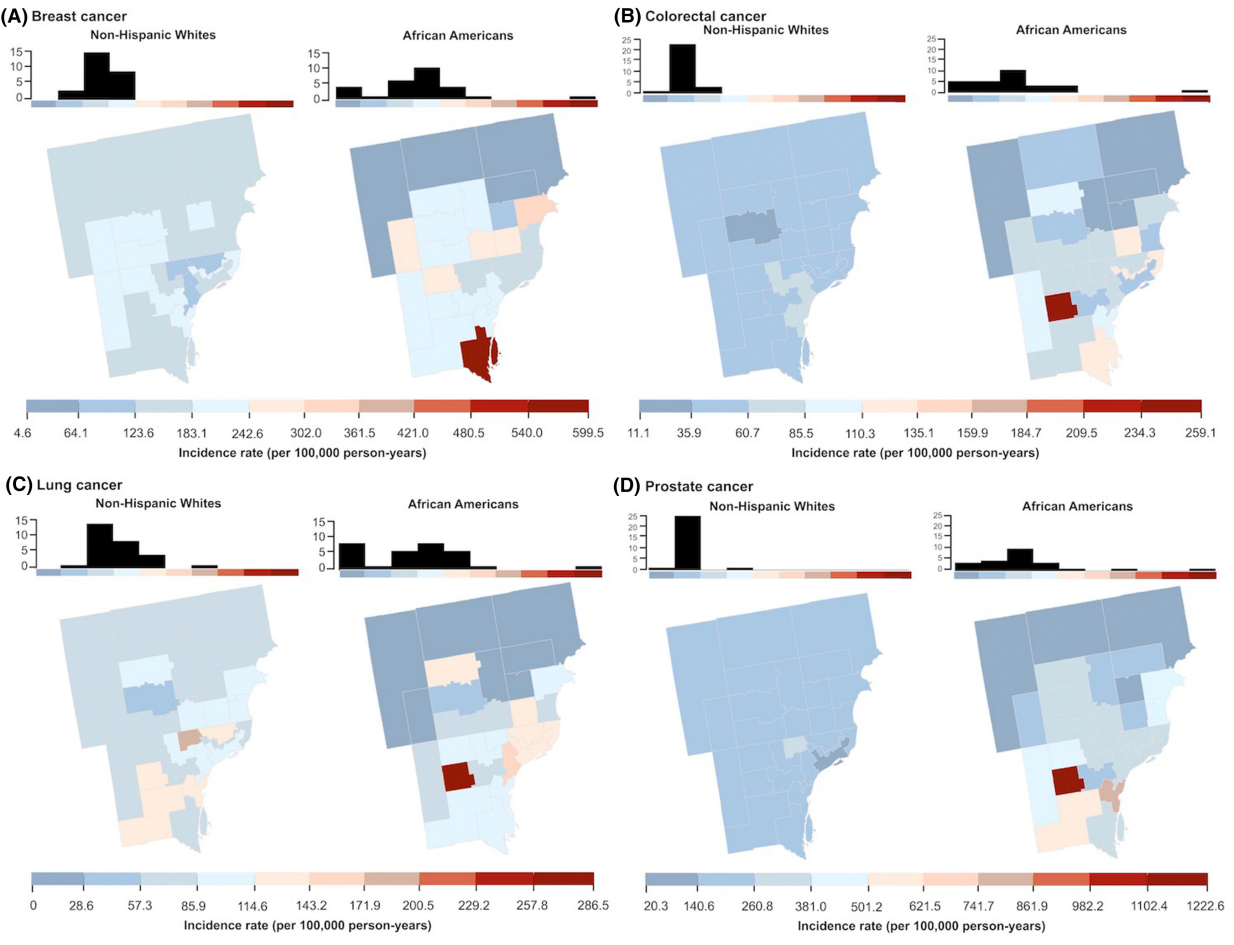
Incidence rates per 100,000 person‐years adjusted for age and sex (colorectal and lung only) for (A) breast, (B) colorectal, (C) lung, and (D) prostate cancer are shown separately for non‐Hispanic Blacks (NHB) and non‐Hispanic Whites (NHW) in each of 27 public use microdata areas (PUMAs) in Wayne, Oakland, and Macomb counties in Michigan. PUMAs are shaded according to incidence rate decile as indicated by the scale in each panel, with darker blue indicating lower incidence rates and darker red indicating higher incidence rates. Cancer site‐specific deciles were calculated with both racial groups combined. Histograms depicting the distribution of PUMAs across incidence rate deciles are shown separately for NHWs and NHBs above the corresponding map.

Cancer incidence rates across the 27 PUMA units in metropolitan Detroit ranged from 4.6 to 1222.6 cases per 100,000 PY across the four cancer sites (Figure 1). Median PUMA‐level cancer incidence was higher among NHBs compared to NHWs for breast (209.1 vs. 174.0 per 100,000 PY, *p* = 0.018), colorectal (70.5 vs. 52.3 per 100,000 PY, *p* = 0.0033), and prostate cancer (327.9 vs. 179.2 per 100,000 PY, *p* = 0.00015). Incidence rates were also more variable for NHB than for NHWs across all cancer sites, where the interquartile ranges (IQR) of PUMA‐level incidence rates were 2.5‐ to 4.5‐fold higher for NHBs compared to NHWs (Breast IQR: 72.1 vs. 28.6 per 100,000 PY; Colorectal IQR: 23.6 vs. 12.4 per 100,000 PY; Lung IQR: 88.2 vs. 37.1 per 100,000 PY; Prostate IQR: 146.4 vs. 32.3 per 100,000 PY, for NHB vs. NHW, respectively).

The majority of cases were diagnosed between 45 and 85 years of age, where women with breast cancer had a lower median age at diagnosis (61 years) and individuals with lung cancer had higher median age at diagnosis (68 years) than individuals with colorectal and prostate cancers (65 years) (*p* < 0.0001) (Table 1). Colorectal and lung cancer cases were approximately evenly distributed among men and women. The majority of cases were non‐Hispanic White (NHW, ~73%), except for triple negative (TN) breast cancer, which had a substantially higher proportion of NHB women (40.3%). The majority of cancer cases lived in areas assigned to ADI quartiles 1 and 2 (Tables 1). At the PUMA unit‐level, the proportion of residents of self‐reported NHB race was highly correlated with ADI quartile (Spearman ρ = 0.79, *p* < 0.0001; Global Moran's *I* = 0.46) (Figure S1). The distribution of the 17 US census factors used to calculate ADI are shown by ADI quartile in Table S1.

ADI was inversely associated with overall breast cancer incidence among NHW and NHB women adjusting for age (NHW: per‐quartile RR = 0.92, 95% CI 0.88–0.96, *p* = 0.00028; NHB: per‐quartile RR = 0.94, 95% CI 0.91–0.98, *p* = 0.0017) (Table 2). This inverse association remained only for HR+/HER2− breast cancer incidence among NHW women (per‐quartile RR = 0.92, 95% CI 0.87–0.96, *p* = 0.00079), HR+/HER2+ (per‐quartile RR = 0.89, 95% CI 0.80–0.98, *p* = 0.024), TN (per‐quartile RR = 0.90, 95% CI 0.82–0.97, *p* = 0.010), and marginally for HR+/HER2− (per‐quartile RR = 0.93, 95% CI 0.88–1.01, *p* = 0.056) breast cancer incidence among NHB women. ADI was also inversely associated with prostate cancer incidence among NHW men (per‐quartile RR = 0.94, 95% CI 0.90–0.97, *p* = 0.00053), but was not significantly associated with prostate cancer incidence among NHB men. Interestingly, while not statistically significant, ADI was associated with an increased risk of prostate cancer among NHB men (per‐quartile RR = 1.06, 95% CI 0.97–1.15). ADI was also significantly associated with increased colorectal and lung cancer incidence among both NHW and NHB individuals, adjusting for age and sex. Among NHWs, colorectal cancer risk was significantly increased only among ADI quartiles 2 and 3 compared to quartile 1. Among NHBs, each increase in ADI quartile was associated with an 11% increase in colorectal cancer risk (95% CI 1.02–1.21, *p* = 0.010). Among NHWs, lung cancer incidence increased 12% for each increase in ADI quartile (95% CI 1.04–1.21, *p* = 0.0028). ADI was more strongly associated with lung cancer incidence among NHBs where each increase in ADI quartile was associated with a 37% increase in risk (95% CI 1.25–1.51, *p* < 0.0001).

We next evaluated the relationship between race and cancer incidence as a first step toward evaluating the role of ADI in this relationship (Table 3). We observed significantly higher cancer incidence rates among NHBs compared to NHWs for TN breast, colorectal, prostate, and lung cancers. NHBs were 11% more likely to be diagnosed with lung cancer (95% CI 1.07–1.16, *p* < 0.0001), 33% more likely to be diagnosed with colorectal cancer (95% CI 1.26–1.40, *p* < 0.0001), 64% more likely to be diagnosed with prostate cancer (95% CI 1.58–1.71, *p* < 0.0001), and nearly twofold more likely to be diagnosed with TN breast cancer (95% CI 1.76, 2.17, *p* < 0.0001) than NHWs controlling for age and sex, when appropriate. NHB women were about 20% less likely to be diagnosed with HR+/HER2− breast cancer (95% CI 0.74–0.83, *p* < 0.0001).

Hypothesizing that inclusion of ADI would attenuate the relationship between race and cancer incidence in these models, we further adjusted for ADI and evaluated changes in the relative risks describing the association between race and cancer incidence. When adjusting for ADI, race‐associated relative risks increased for TN breast and prostate cancers, with ADI accounting for a statistically significant 2.3% increase in the relationship between race and prostate cancer risk (95% CI 1.1–3.8%, *p* < 0.0001). In contrast, race‐associated relative risks decreased for HR+/HER2− breast, colorectal, and lung cancers when adjusting for ADI. ADI attenuated the effect of race by 8.5% (95% CI 4.1–16.6%, *p* < 0.0001) for HR+/HER2− breast cancer incidence and 7.3% (95% CI 3.7–12.8%, *p* < 0.0001) for colorectal cancer incidence. ADI did not significantly attenuate the relationship between race and lung cancer incidence (proportion attenuated: −117%, 95% CI −153–131%, *p* = 0.31).

**FIGURE 2:**
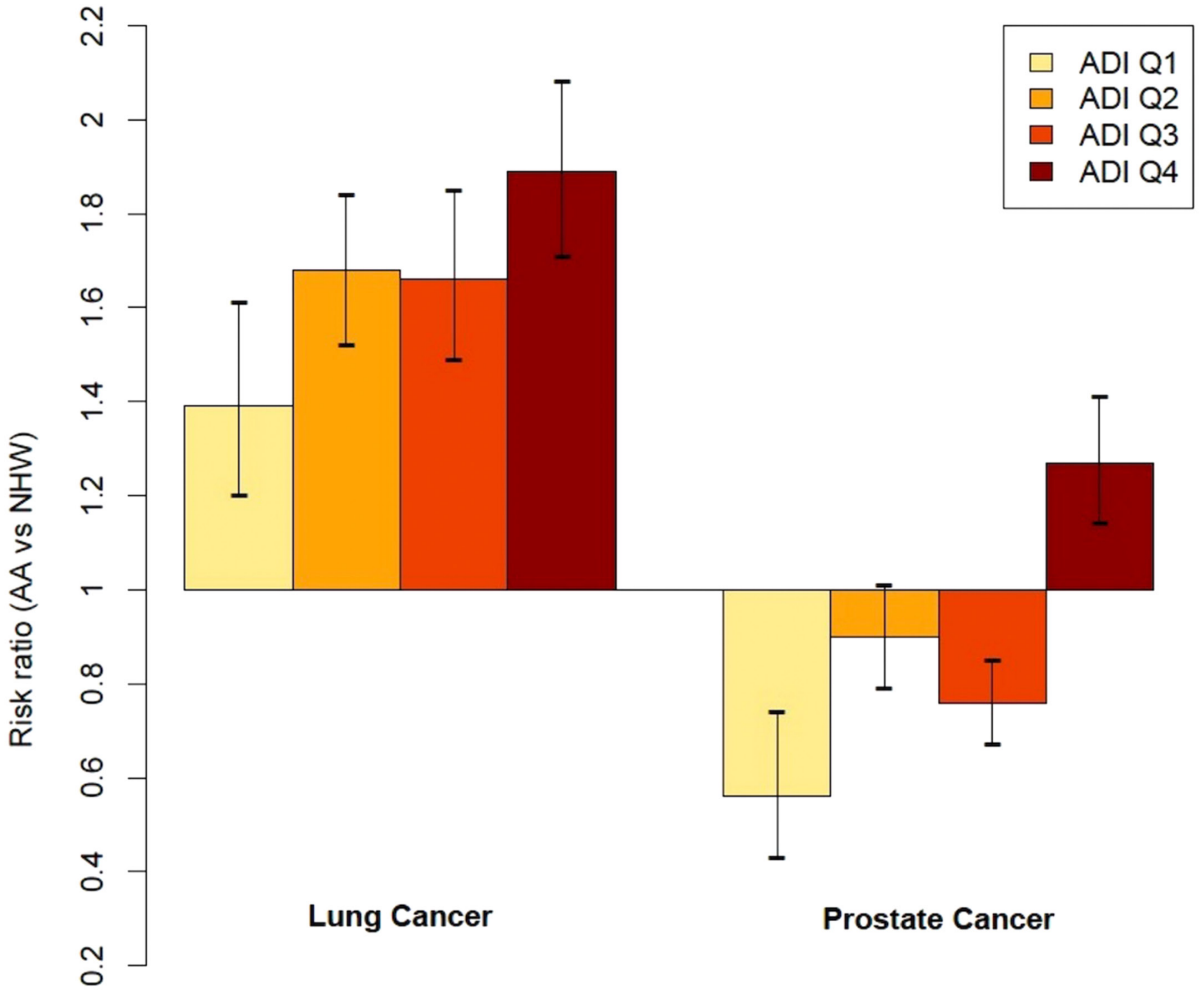
Risk ratios and 95% confidence intervals for the associations between race and cancer incidence are shown for lung and prostate cancer separately by ADI quartile. Height of bars corresponds to the magnitude of the risk ratio. 95% Confidence intervals are depicted by vertical black lines capped with horizontal dashes.

In a post hoc analysis, we also evaluated interactions between ADI and race. Statistically significant interactions between race and ADI were observed for lung (interaction RR = 1.22, *p* < 0.0001) and prostate (interaction RR = 1.09, *p* = 0.00092) cancer (Figure 2). For lung cancer, the strength of the association between race and incidence increased as ADI quartile increased (Q1: RR = 1.39, 95% CI 1.20–1.61; Q2: RR = 1.68, 95% CI 1.52–1.84; Q3: RR = 1.66, 95% CI 1.49–1.85; Q4: RR = 1.89, 95% CI 1.71–2.08). Interestingly, NHB men were less likely to be diagnosed with prostate cancer compared to NHW men if they lived in regions with lower deprivation (Q1: RR = 0.56, 95% CI 0.43–0.74; Q2: RR = 0.90, 95% CI 0.79–1.01; Q3: RR = 0.76, 95% CI 0.67–0.85), but were more likely to be diagnosed with prostate cancer if they lived in regions with the highest deprivation levels (Q4: RR = 1.27, 95% CI 1.14–1.41).

## 4 DISCUSSION

The social construct of race in the United States was developed as a justification for the dependence of the early American economic system on forced labor, particularly the enslavement of Africans and people of African descent.^29^ Centuries of pervasive systemic and institutional racism have resulted in racially segregated neighborhoods with substantial differences in neighborhood quality and resources, with clear effects on human health including cancer.^30^ Metropolitan Detroit remains one of the most racially segregated regions in the country,^31^ and it is in this context that we hypothesized not only that neighborhood‐level social disadvantage would be associated with cancer incidence, but that it would capture many of the adverse conditions that lead to increased cancer incidence among NHBs compared to NHWs. Indeed, we found that ADI differentially affected cancer risk by race to varying degrees across cancer sites and that ADI attenuated the relationship between race and both HR+/HER2− breast cancer and colorectal cancer incidence. Further, we found that associations between race and both lung and prostate cancer incidence varied by ADI. To understand these associations, it is important to recognize the many ways in which segregation and structural racism have resulted in disproportionate exposure to adverse conditions that affect the health of disadvantaged, and largely NHB, neighborhood residents. These include neighborhood‐level characteristics such as pollution and lack of health‐promoting resources that directly influence health, as well as the indirect effects of neighborhood through individual‐level health behaviors and socioeconomic resources.

Location‐based mortgage lending bias (i.e., “redlining”), in which mortgage lenders flagged communities with large NHB populations as hazardous for investment, resulted in segregation of NHBs into neighborhoods with many characteristics that have been shown to chronically impair health. Neighborhood segregation is associated with higher air pollution levels and exposure to fine particulate matter (PM_2.5_),^32^, ^33^ due to intentional routing of highways and disproportionate placement of landfills, incinerators, airports, and bus depots in NHB and other minority neighborhoods.^30^, ^34^ PM_2.5_ has been associated with increased risk of lung, breast, and colon cancers, independent of cigarette smoking,^35^, ^36^, ^37^ while nitrogen dioxide and other measures of traffic exposure have been associated with increased lung, breast, and prostate cancer risk.^38^, ^39^, ^40^ Further, it has been suggested that residents of disadvantaged neighborhoods have increased susceptibility to the adverse health effects of air pollution due to poorer baseline health status and lack of resources to deal with pollution.^41^

Neighborhood deprivation further affects health through a lack of access to high quality healthcare and sufficient resources to promote health.^42^, ^43^ We observed that higher ADI was associated with lower rates of breast and prostate cancer, which is likely to be partially reflecting differences in access to cancer screening services such as mammography, digital rectal exams, and prostate‐specific antigen testing. Women of higher SES are more likely to report physician recommendations and uptake of mammography,^44^ although some studies have suggested that white women experience more pronounced increases in the likelihood of being screened associated with SES than NHB women.^45^ This is consistent with our observation that the magnitude of the ADI association with breast cancer incidence was stronger for NHW than NHB women. Similarly, residents of socioeconomically disadvantaged neighborhoods are less likely to undergo prostate and colorectal cancer screening.^46^, ^47^, ^48^, ^49^ Triple negative breast cancer incidence has also been linked to area‐level density of obstetrician/gynecologists.^50^ Further compounding the effects of the lack of high‐quality healthcare, smoking‐related inequities in disadvantaged neighborhoods are exacerbated by increased availability of cheaper cigarettes and lack of access to effective smoking cessation programs.^51^, ^52^, ^53^, ^54^

Deprived neighborhoods also affect behavioral cancer risk factors such as physical activity, diet, and obesity through lack of access to supermarkets and safe spaces for recreation/physical activity, which is consistent with our observation that higher ADI was associated with higher rates of colorectal and lung cancer.^17^, ^18^ The relationship between neighborhood and healthy food consumption is complex, and has been found to be a product of diversity in food choices, relative availability of supermarkets versus convenience stores, and changes in neighborhood attributes over time.^54^, ^55^, ^56^, ^57^ The built environment seems to have a stronger relationship with physical activity and sedentary behavior, with variability depending on age and gender.^56^, ^58^, ^59^ Importantly, research on neighborhood deprivation and health behaviors such as physical activity and increased fruit/vegetable consumption suggests that interventions and policies that improve environments and reduce deprivation have substantial potential to improve these health behaviors.^60^

Our observed variation in the effect of ADI on racial differences in cancer incidence may partially reflect that there are racial differences in individuals' experiences of area‐level socioeconomic deprivation. A recent “neighborhood wide association study” found substantial differences between Black and White men in the roles of housing, education, employment, transportation, and income on the diagnosis of advanced prostate cancer.^61^ These differences are further driven by factors such as increased discrimination, social isolation, crime, and limited perceived safety at the neighborhood level.^62^, ^63^, ^64^, ^65^, ^66^ These factors have been associated with increased levels of chronic stress and systemic inflammation.^67^, ^68^, ^69^ Chronic stress hormones, resulting from perturbation of the hypothalamic–pituitary–adrenal axis and the sympathetic nervous system, promote tumorigenesis through multiple mechanisms.^70^ Chronic stress is also associated with changes in immune function and inflammatory response, which is significant because chronic inflammation and immunosenescence also have demonstrated roles in tumorigenesis.^70^, ^71^, ^72^

There were several strengths of our study. First, this was a large analysis using data from the population‐based MDCSS registry, increasing both the generalizability and validity of our results. Second, metropolitan Detroit is a racially diverse region, allowing us to have sufficient sample sizes for both NHW and NHB residents to calculate robust measures of incidence and relative risk. Third, we utilized a validated factor‐based index of area‐level deprivation capturing census indicators of poverty, education, housing, and employment. One limitation of the study is that our analyses were limited to relatively large geographical regions due to the nature of publicly available population count data, which also required that we indirectly calculate ADI for these regions using weighted medians of census‐tract level data. Second, we restricted our analyses to include only NHW and NHB residents because other racial/ethnic groups are far less prevalent in metropolitan Detroit, particularly when stratifying by PUMA region. Third, we were unable to obtain length of residence at current address as well as residential addresses prior to cancer diagnosis, which could be relevant when considering ADI as an etiologic factor. Neighborhood socioeconomic deprivation is likely to play a role throughout the years‐ or decades‐long process of tumorigenesis. These associations may be weakened or undetectable only using residential ADI at the time of cancer diagnosis. Finally, we had limited individual‐level data due to the nature of the study design, allowing us to control for sex and age and unable to account for individual SES factors, insurance coverage, and health behaviors, which may in fact partially mediate the association between ADI and cancer incidence. Future studies explicitly designed to evaluate the direct effects of ADI as well as its indirect effects through individual‐level behavioral and socioeconomic factors are needed to better understand the mechanisms through which ADI influences cancer incidence.

In summary, area‐level socioeconomic disadvantage is associated with risk of common cancers in a racially diverse population, and it plays a role in racial differences in cancer incidence. This highlights the importance of evaluating neighborhood context in understanding cancer risk and racial disparities in cancer, especially when considering etiologic factors that are associated with race and tumor aggressiveness. Future work to obtain information at the individual‐ and area‐levels will provide valuable insight on how best to focus efforts at cancer prevention and reduction of racial disparities in cancer risk. Most cancer prevention efforts to date, including those designed to reduce health disparities, have focused on changing behavior at the individual, interpersonal, or community levels; however, these have not translated to sustainable, long‐term reductions in racial cancer disparities in part because they do not address the structural barriers that prevent meaningful behavior change, such as lack of access to high quality healthcare, recreational spaces for physical activity, and low‐cost healthy food options.^73^, ^74^ A better understanding of the mechanisms—both direct and indirect—by which area‐level socioeconomic disadvantage affects cancer incidence would help inform scientific and policy‐level discussions about the benefit of upstream structural interventions to make meaningful improvements in racial health equity.

## AUTHOR CONTRIBUTIONS

**Kristen S. Purrington:** Conceptualization (lead); data curation (lead); formal analysis (lead); writing – original draft (lead); writing – review and editing (lead). **Theresa A. Hastert:** Conceptualization (supporting); methodology (supporting); writing – review and editing (equal). **KC Madhav:** Data curation (supporting); formal analysis (supporting); writing – review and editing (equal). **Mrudula Nair:** Data curation (supporting); writing – review and editing (equal). **Natalie Snider:** Conceptualization (supporting); formal analysis (supporting); writing – review and editing (equal). **Julie J. Ruterbusch:** Data curation (equal); formal analysis (supporting); writing – review and editing (equal). **Ann G. Schwartz:** Data curation (supporting); writing – review and editing (equal). **Elena M. Stoffel:** Conceptualization (supporting); writing – review and editing (equal). **Edward S. Peters:** Conceptualization (supporting); data curation (supporting); writing – review and editing (equal). **Laura S. Rozek:** Conceptualization (equal); data curation (supporting); writing – review and editing (supporting).

## FUNDING INFORMATION

This work was supported, in part, by NCI R01CA259420, and the Epidemiology Research Core and NIH Center Grant P30CA022453 to the Karmanos Cancer Institute at Wayne State University for conduct of the study. This work was also supported in part by contract HHSN261201300011I.

## CONFLICT OF INTEREST STATEMENT

The authors have no conflict of interest to declare.

## Supporting information


**Figure S1.** **Table S1**

## Data Availability

The United States census data that support the findings of this study are available at https://usa.ipums.org/usa/ (Sample 201,603), https://gis‐michigan.opendata.arcgis.com/datasets/ (2010 Census Tracts v17a), and https://data.census.gov/mdat/#/ (Dataset: ACS 5‐Year Estimates 5‐Year Estimates – Public Use Microdata Sample; Vintage: 2013). Metropolitan Detroit cancer data are available by request from the Metropolitan Detroit Cancer Surveillance System with appropriate Institutional Review Board approvals.
