# Supplementary material for: The role of area‐level socioeconomic disadvantage in racial disparities in cancer incidence in metropolitan Detroit
Source: Cancer Med. 2023 May 15;12(13):14623–35. doi: 10.1002/cam4.6065 (PMC10358249; doi:10.1002/cam4.6065)
Supplement: Supplementary file 1 — Figure S1. Table S1 [file CAM4-12-14623-s001.docx]

**Figure S1. Distributions of ADI and the proportions of residents of self-reported AA race in metropolitan Detroit**
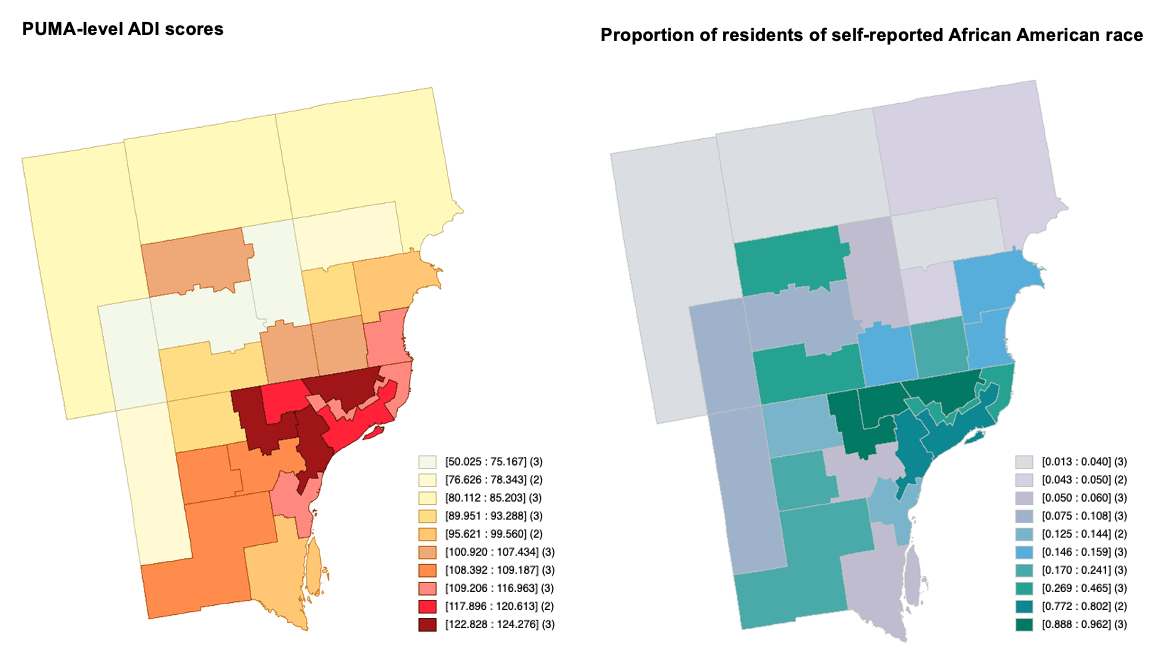
 Maps are shown depicting (a) ADI scores and (b) the proportion of residents of self-reported African American race for each of 27 public use microdata areas in Wayne, Oakland, and Macomb counties in Michigan. (a) Lighter yellow indicates lower ADI and darker red indicates higher ADI. (b) Lighter purple indicates lower proportions and darker green indicates higher proportions of AA residents.

| **Table S1. Distribution of 17 US census indicators by ADI quartile** | |  |  |  |
| --- | --- | --- | --- | --- |
|  | **ADI Q1** | **ADI Q2** | **ADI Q3** | **ADI Q4** |
| **Census indicator** | **Median (IQR)** | **Median (IQR)** | **Median (IQR)** | **Median (IQR)** |
| **Income** |  |  |  |  |
| Median family income, $ | 87,787 (25,653.5) | 63,750 (12,468.5) | 50,250 (10,002) | 32,237 (14,074.5) |
| Income disparity | 0.61 (0.49) | 0.96 (0.39) | 1.27 (0.35) | 1.86 (0.51) |
| Families below poverty level, % | 3.5% (4.0%) | 7.3% (5.5%) | 12.8% (7.5%) | 30.9% (19.9%) |
| Population below 150% of the poverty threshold, % | 10.5% (8.0%) | 19.4% (9.6%) | 28.6% (11.1%) | 51.9% (21.2%) |
| **Housing characteristics** |  |  |  |  |
| Single-parent households with children aged <18y, % | 18.5% (13.2%) | 28.1% (14.9%) | 36.0% (17.4%) | 65.2% (29.1%) |
| Households without a motor vehicle, % | 2.5% (3.5%) | 3.9% (4.3%) | 5.9% (5.6%) | 17.2% (14.9%) |
| Households without a telephone, % | 1.4% (1.9%) | 2.2% (2.0%) | 2.7% (2.4%) | 3.7% (4.1%) |
| Occupied housing units without complete plumbing, % | 0.0% (0.0%) | 0.0% (0.5%) | 0.0% (0.5%) | 0.0% (1.1%) |
| Owner occupied housing units, % | 87.9% (17.6%) | 82.6% (19.2%) | 77.7% (21.5%) | 55.1% (21.6%) |
| Households with more than 1 person per room, % | 0.5% (1.2%) | 0.9% (1.6%) | 1.4% (2.2%) | 2.1% (3.6%) |
| Median month mortgage, $ | 1684 (402) | 1291 (221) | 1102.5 (199) | 994 (225) |
| Median gross rent, $ | 960 (440) | 759 (200) | 705.5 (212) | 737 (214) |
| Median home value, $ | 189,150 (64,400) | 127,000 (25,250) | 91,450 (19,100) | 52,650 (24,500) |
| **Employment** |  |  |  |  |
| Employed persons aged ≥16y in white-collar occupations, % | 46.8% (16.7%) | 32.1% (10.2%) | 26.0% (8.7%) | 18.7% (11.7%) |
| Civilian labor force population aged ≥16 unemployed, % | 7.9% (3.9%) | 10.4% (4.8%) | 13.3% (5.5%) | 23.7% (14.2%) |
| **Education** |  |  |  |  |
| Population aged ≥25y with <9 years education, % | 1.3% (1.6%) | 2.2% (2.2%) | 3.3% (2.6%) | 4.7% (4.4%) |
| Population aged ≥25y with at least a high school diploma, % | 95.4% (3.9%) | 91.5% (5.2%) | 87.6% (5.7%) | 79.8% (12.2%) |
